# Supplementary material for: Interference of Pseudomonas aeruginosa Virulence Factors by Different Extracts from Inula Species
Source: Pharmaceuticals (Basel). 2025 Nov 29;18(12):1824. doi: 10.3390/ph18121824 (PMC12735715; doi:10.3390/ph18121824)
Supplement: Supplementary file 1 [file pharmaceuticals-18-01824-s001.zip › pharmaceuticals-3976389-supplementary.pdf]

SUPPLEMENTARY MATERIAL

Table S1: Compounds identified in chloroform and methanol extracts from *Inula britannica, I. bifrons, I. helenium* and *I. spiraeifolia*

	I. britannica	*I. bifrons*	*I. helenium*	*I. spiraeifolia*
**Chloroform extract**	**IBr1** [30]	**IB1** [31]	**IH1** [in this study]	**ISp1** [in this study]
Sesquiterpenoids and sesquiterpene lactones	Britannin*, Gaillardin*, 11,13-Dihydro inuchinenolide B*, Pulchellin C*, Ivalin*	Costic Acid, Isoalantolactone, Isotelekin, 4α,5α-Epoxy-10,14-dihydro-1-*epi*-inuviscolide, Inuviscolide, 1-*epi*-Inuviscolide	4α,5α-Epoxy-10,14-dihydro-1-*epi*-inuviscolide	-
Diterpenoids	-	*ent*-15α-(3-Methylbutanoyloxy)-16-kauren-19-oic acid, *ent*-15α-(3-Methylpentanoyloxy)-kaur-16-en-19-oic acid, 7β-Acetoxy-*ent*-kaur-16-en-19-oic acid	-	-
Triterpenoids and sterols	β-Amyrin, 16β-Hydroxylupeol palmitate, Maniladiol palmitate, Faradiol palmitate; β-Sitosterol	β-Amyrin, β-Amyrin acetate, β-Amyrin palmitate; 16β-Hydroxylupeol palmitate; β-Sitosterol	β-Amyrin, Taraxasterol, Lupeol and Ψ-Taraxasterol and their acetates and palmitates 16β-Hydroxylupeol palmitate Maniladiol palmitate Faradiol palmitate β-Sitosterol	β-Amyrin, Taraxasterol, Lupeol and Ψ-Taraxasterol and their acetates and palmitates 16β-Hydroxylupeol palmitate Maniladiol palmitate Faradiol palmitate β-Sitosterol
**Methanol extract**	**IBr2** [30, 33]	**IB2** [18]	**IH2** [in this study]	**ISp2** [in this study]
Caffeoylquinic acids	Chlorogenic acid, 1,5-,3,5-, 4,5- and 3,4-dicaffeoylquinic acids	Chlorogenic acid, 1,5-, 3,5-, 4,5- and 3,4-dicaffeoylquinic acids	Chlorogenic acid, 1,5- and 3,5-dicaffeoylquinic acids	Chlorogenic acid, 1,5-, 3,5-, 4,5- and 3,4- Dicaffeoylquinic acids
Flavonoids	Luteolin, Quercetin, Luteolin-7-*O*-glucoside	-	Quercetin, Isoquercetin	Luteolin, Kaempferol, Apigenin, Quercetin, Rutin, Hyperoside, Isoquercetin

*These compounds present in sesquiterpene enriched fraction IBr1-SL.

Fractionation of *I. helenium* and *I. spiraeifolia* chloroform extracts and identification of the main compounds.

A portion of *I. helenium* (**IH1**) chloroform extract (0.660 g) was subjected to a column chromatography (CC) on Silica gel, using Hexane/Ethyl acetate mixtures with increasing polarity (from 10:1 to 0:1). The separation process was monitored by TLC (Silica gel, Hexane/Et_2O, 2:1) and 8 fractions were collected: **IH1-1** (102 mg), **IH1-2** (172 mg), **IH1-3** (147 mg), **IH1-4** (85 mg,), **IH1-5** (17 mg), **IH-1-6** (29 mg), **IH1-7** (132 mg) and **IH1-8** (230 mg). A portion of fr. **IH1-1** (20 mg) was hydrolysed under alkaline conditions. GC-MS analysis of the obtained free alcohols and methylated fatty acids allowed the identification of β-amyrin, lupeol, taraxasterol, and Ψ-taraxasterol palmitates. GC-MS analysis of fr. **IH1-2** led to identification of β-amyrin, lupeol, taraxasterol, and Ψ-taraxasterol acetates. CC (Hexane/EtOAc, 6:1) of fr. **IH1-3** and further purification by prep. TLC (Hexane/EtOAc, 6:1) afforded 16-hydroxylupeol-*O*-palmitate (2.5 mg), maniladiol palmitate (3.8 mg) and faradiol palmitate (3.5 mg). CC (Hexane/EtOAc, 6:1) of **IH1-4** (85 mg) afforded 16-hydroxylupeol-*O*-palmitate (15.8 mg), β-amyrin (6.7 mg) and β-sitosterol (2.1 mg). GC/MS analysis of fr. **IH1-5** led to identification of β-amyrin, lupeol, taraxasterol, and Ψ-taraxasterol. Prep. TLC (Hexane/EtOAc, 5:1) of a portion of **IH1-7** (20 mg) afforded 4α,5α-epoxy-10α,14-dihdyro-1-*epi*-inuviscolide (8 mg).

CC of *I. spiraeifolia* (**ISp1**) chloroform extract (0.450 g) afforded 6 fractions: **ISp1-1** (225 mg), **ISp1-2** (60 mg), **ISp1-3** (74 mg), **ISp1-4** (23 mg), **ISp1-5** (21 mg), and **ISp1-6** (32 mg). A portion of fr. **ISp1-1** (20 mg) was hydrolysed under alkaline conditions. GC-MS analysis of the obtained free alcohols and methylated fatty acids allowed the identification of β-amyrin, lupeol, taraxasterol, and Ψ-taraxasterol palmitates. GC-MS analysis of fr. **ISp1-2** led to identification of β-amyrin, lupeol, taraxasterol, and Ψ-taraxasterol acetates. Prep. TLC (Hexane/EtOAc, 6:1) of a portion of **ISp1-3** (15 mg) afforded 16-hydroxylupeol-*O*-palmitate (1.5 mg), maniladiol palmitate (3.2 mg) and faradiol palmitate (2.5 mg). GC-MS analysis of fr. **ISp1-4** led to identification of β-amyrin, lupeol, taraxasterol, and Ψ-taraxasterol. Fr. **ISp1-5** contained mainly β-sitosterol (TLC comparison with authentic standard).

Fractionation of *I. helenium* and *I. spiraeifolia* methanol extracts and identification of the main compounds

A portion of methanol extract from *I. helenium* (**IH2**, 0.150 g), *I. spiraeifolia* (**ISp2**, 0.320 g) was dissolved in methanol (10 mL) and centrifuged at 5800 rpm in order to remove insoluble parts. The clear methanol solution was concentrated up to 5 mL and subjected to a Sephadex LH-20 column (equilibrated with 50 mL of methanol) to three main fractions **A**-**C**. TLC comparison of fractions **IH2-B, ISp2-B** and **IB2-B** (Silica gel 60 F_{254}, Toluene:EtOAc/HCOOH/H_2O, 5:100:10:10, spraying with NP reagent (1% diphenylboronic acid 2-aminoethyl ester in ethyl acetate and UV visualization at 366 nm) with standards showed the presence of chlorogenic acid, 1,5-, 3,5-, 3,4- and 4,5-dicaffeoylquinic acids (blue fluorescence) in **ISp2-B** and **IB2-B** and chlorogenic acid, 1,5- and 3,5-dicaffeoylquinic acids in **IH2-B.**

Prep. TLC (RP-18, MeOH/H_2O, 1:1) of fraction **IH2-C** (25 mg) led to isolation of quercetin (2.5 mg) and isoquercetin (2.6 mg).

MPLC (LiChroprep RP-18, increasing concertation of MeOH in H_2O, from 20 to 80%) of **ISp2-C** (90 mg) afforded rutin (6.9 mg), hyperoside (8.0 mg), isoquercetin (6.1 mg), quercetin (3.2 mg), luteolin (2.8 mg), kaempferol (3.5 mg) and apigenin (1.2 mg).

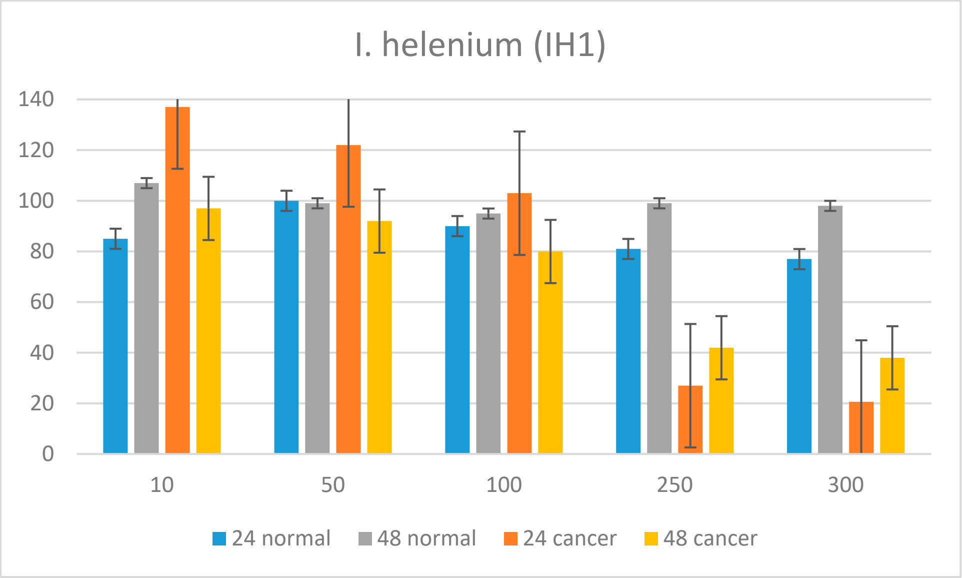

Figure S1. Cytotoxicity of plant extracts on human cells

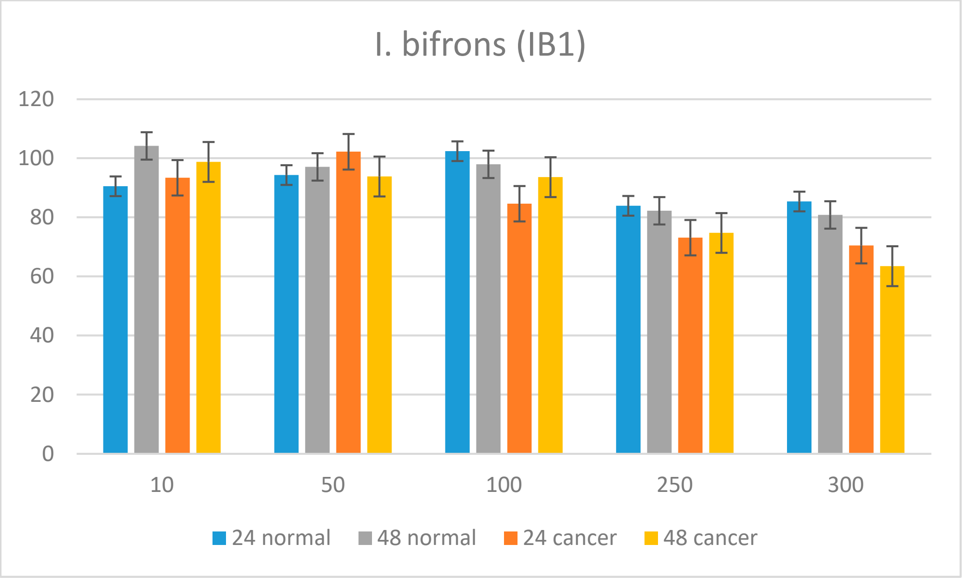

Figure S2. Cytotoxicity of chloroform extracts of *I. helenium* on normal (HFF) and cancer (HepG2 human hepatocarcinoma) after 24 and 48 h treatment. Mean values with SE bars.

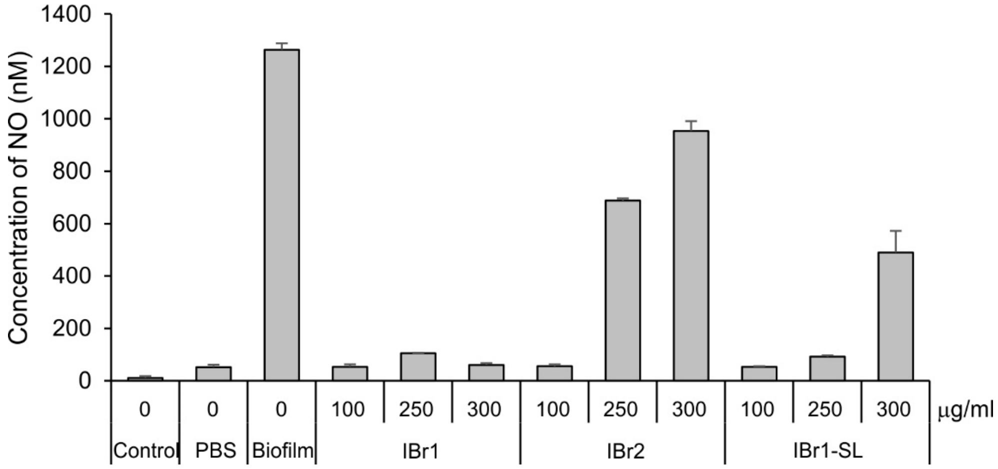

Figure S3. A dose-dependent effect of IBr extracts on NO release by murine skin explants with *P. aeruginosa* biofilm. The data represent a mean± SD of SD of n=3 repeats per group.

References:

[30]. Ivanova, V., Trendafilova, A., Todorova, M., Danova, K. and Dimitrov, D., 2017. Phytochemical profile of Inula britannica from Bulgaria. *Natural product communications*, *12*(2), 153-154. https://doi.org/10.1177/1934578X1701200201

[33]. Ivanova, V., Todorova, M., Rangelov, M., Aneva, I. and Trendafilova, A., 2020. Phenolic content and antioxidant capacity of Inula britannica from different habitats in Bulgaria. *Bulg. Chem. Commun*, *52*, pp.168-173.

[31]. Ivanova, V., Todorova, M., Aneva, I., Nedialkov, P. and Trendafilova, A., 2020. A new ent-kaur-16-en-19-oic acid from the aerial parts of Inula bifrons. *Biochemical Systematics and Ecology*, *93*,_p.104141. https://doi.org/10.1016/j.bse.2020.104141

[18]. Trendafilova, A., Ivanova, V., Rangelov, M., Todorova, M., Ozek, G., Yur, S., Ozek, T., Aneva, I., Veleva, R., Moskova-Doumanova, V. and Doumanov, J., 2020. Caffeoylquinic acids, cytotoxic, antioxidant, acetylcholinesterase and tyrosinase enzyme inhibitory activities of six Inula species from Bulgaria. *Chemistry & Biodiversity*, *17*(4), e2000051. https://doi.org/10.1002/cbdv.202000051

Spectral data of the compounds identified in *I. helenium* and *I. spiraeifolia* extracts

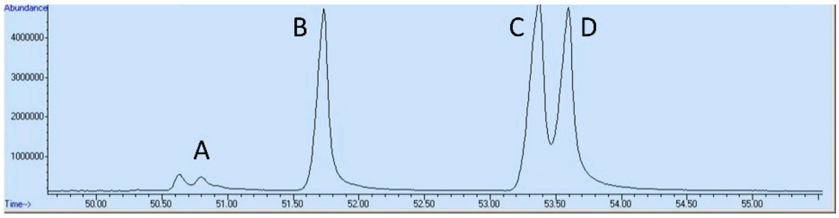

Figure S4. TIC of fr. **ISp1-2** (triterpene acetates)

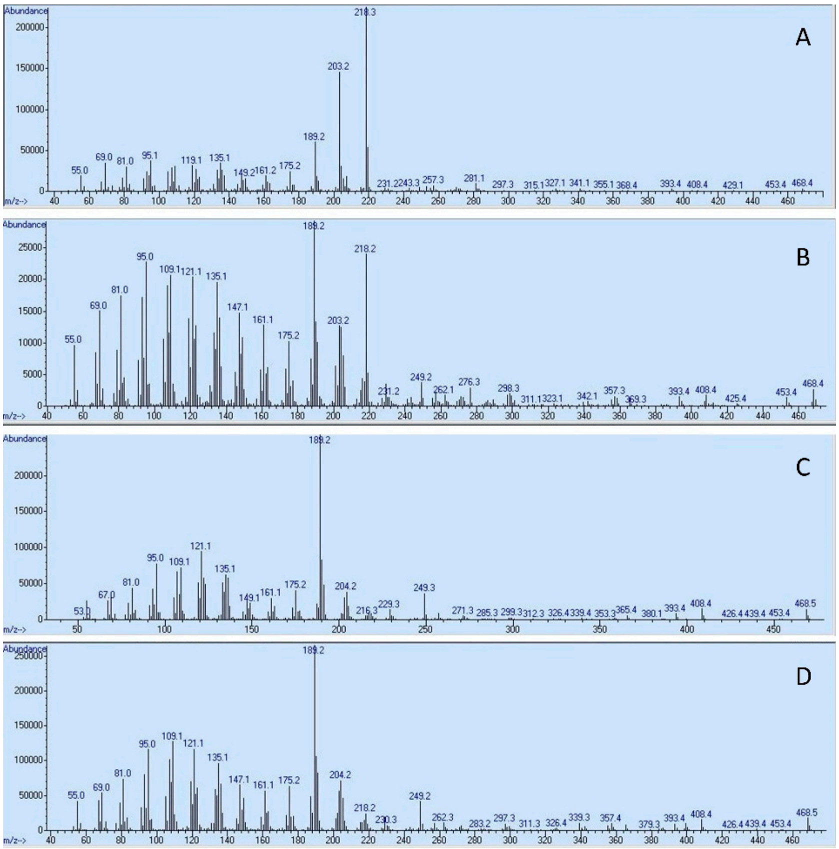

Figure S5. Mass-spectra of β-amyrin acetate (A), lupeol acetate (B), taraxasterol acetate (C) and ψ-taraxasterol acetate (D)

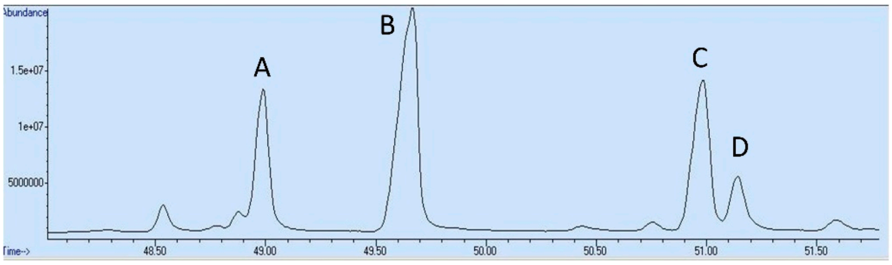

Figure S6. TIC of fr. **IH1-5**(triterpene alcohols)

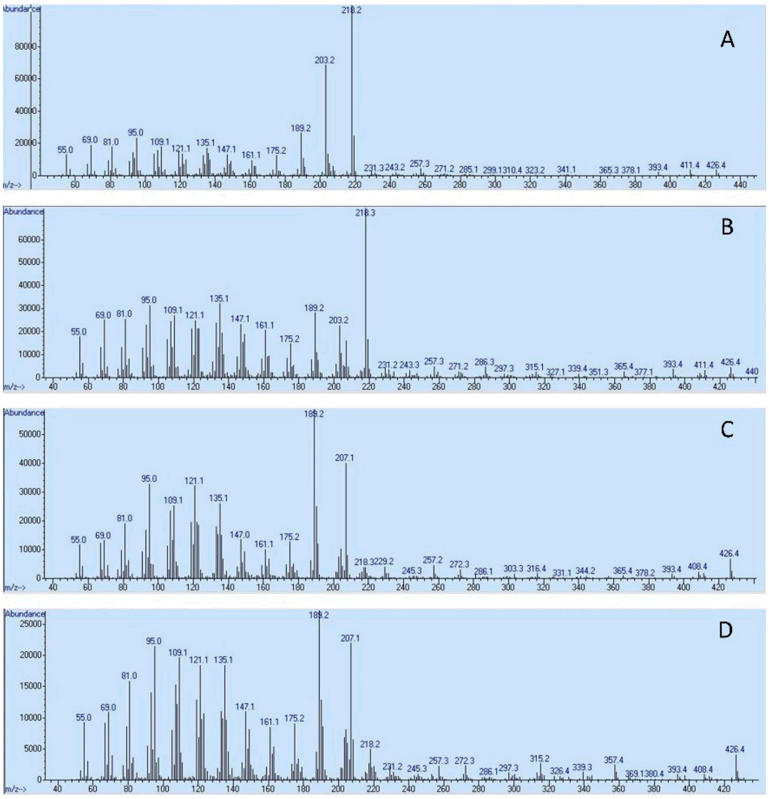

Figure S7. Mass-spectra of β-amyrin (A), lupeol (B), taraxasterol (C) and ψ-taraxasterol (D)

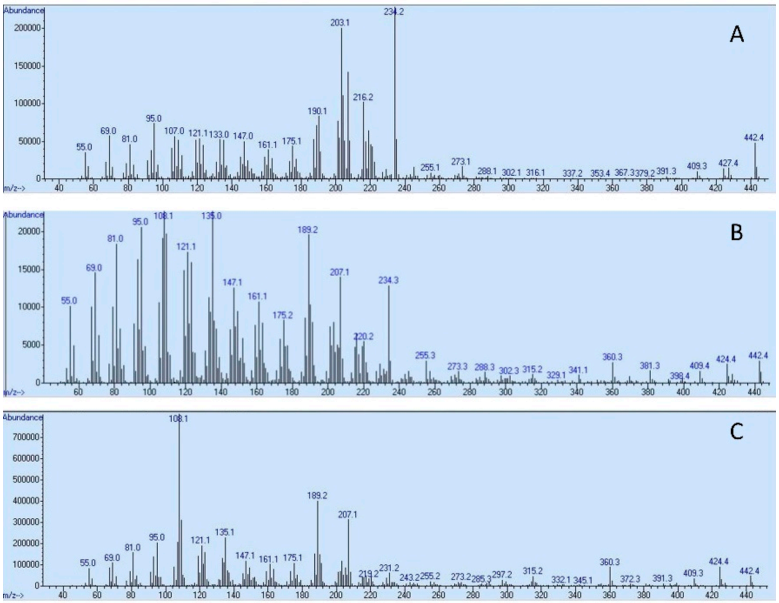

Figure S8. Mass-spectra of maniladiol (A), 16-hydroxylupeol (B) and faradiol (C)

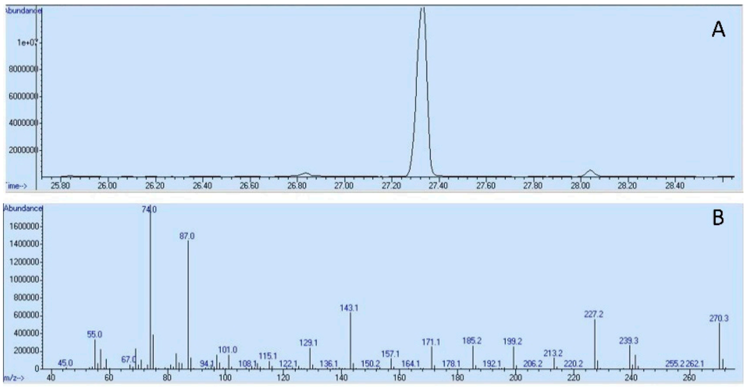

Figure S9. TIC (A) and mass-spectrum (B) of methyl palmitate

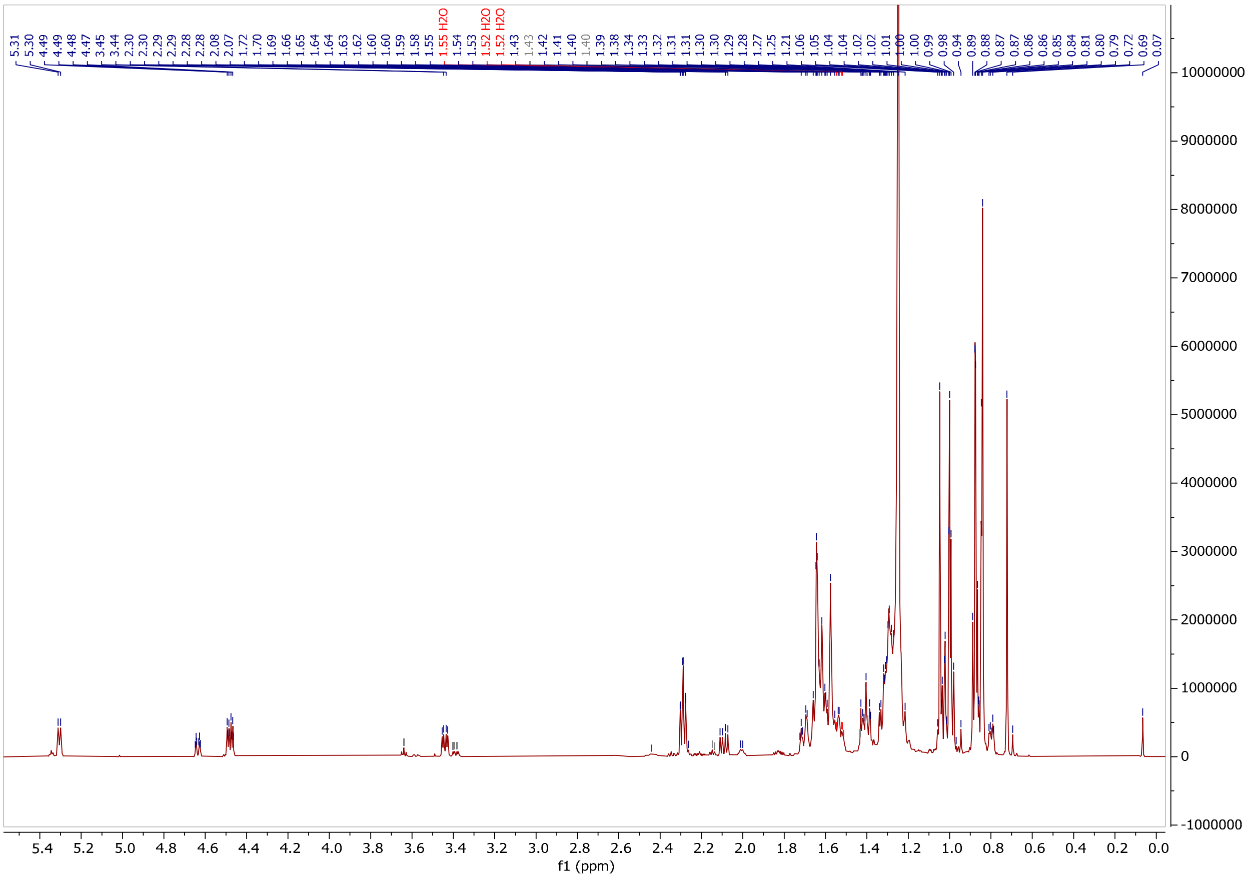

Figure S10. ^{1}H NMR ($CDCl_3$, 600 MHz) of faradiol palmitate

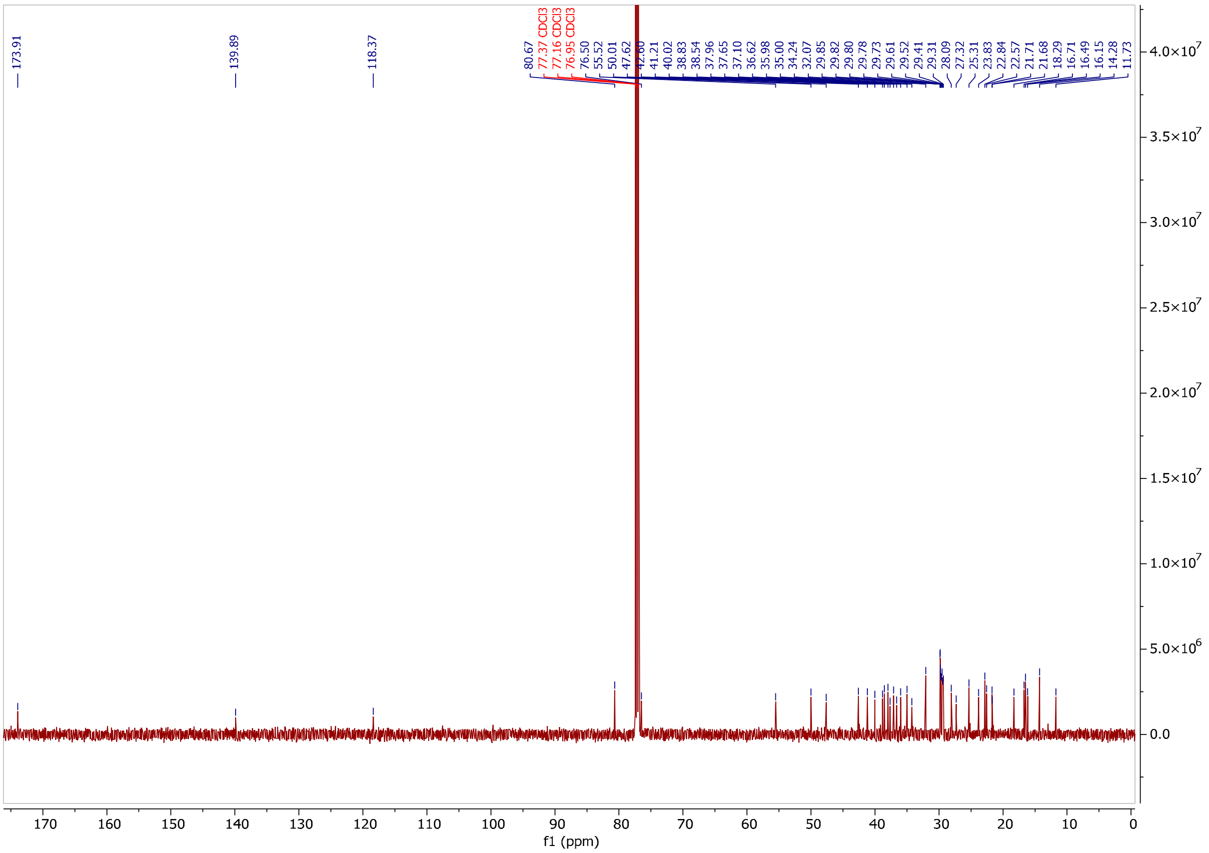

Figure S11. ^{13}C NMR ($CDCl_3$, 150 MHz) of faradiol palmitate

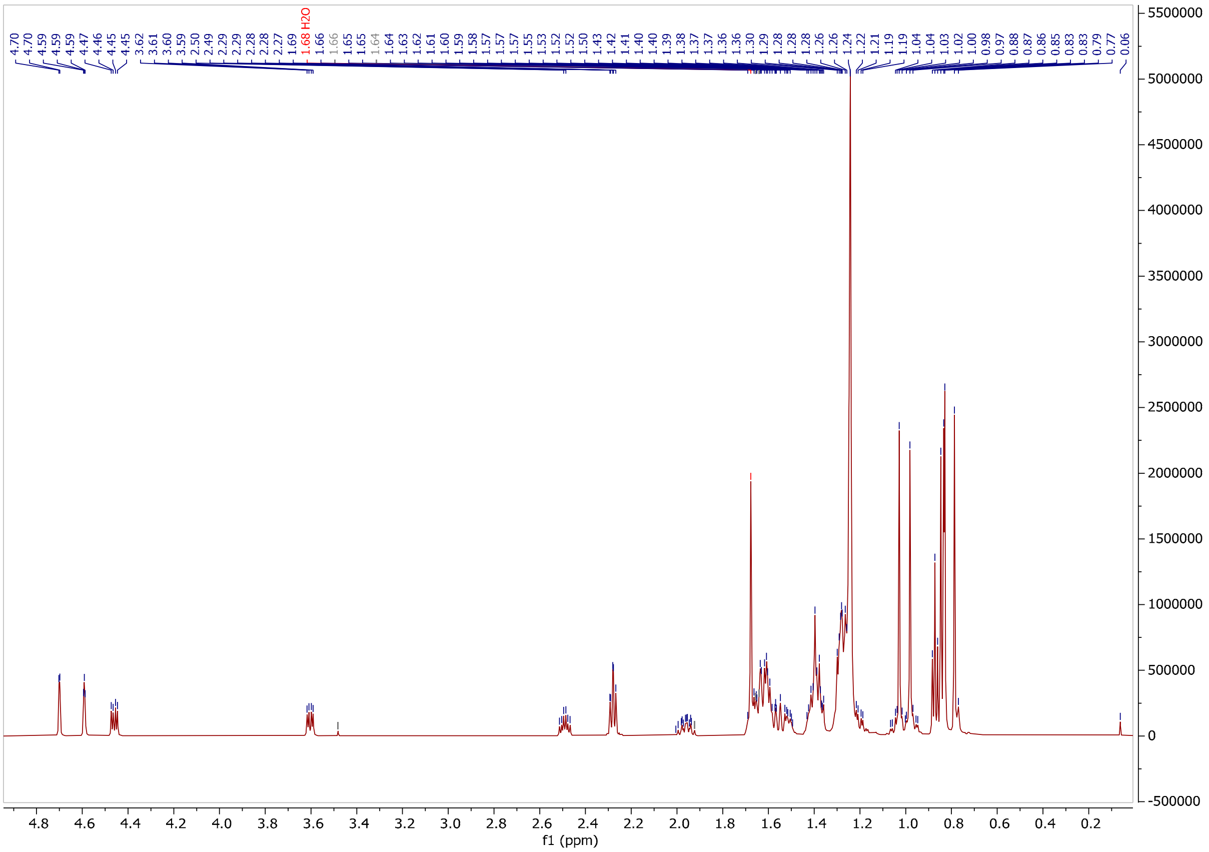

Figure S12. ^{1}H NMR ($CDCl_3$, 600 MHz) of 16-hydroxylupeol-*O*-palmitate

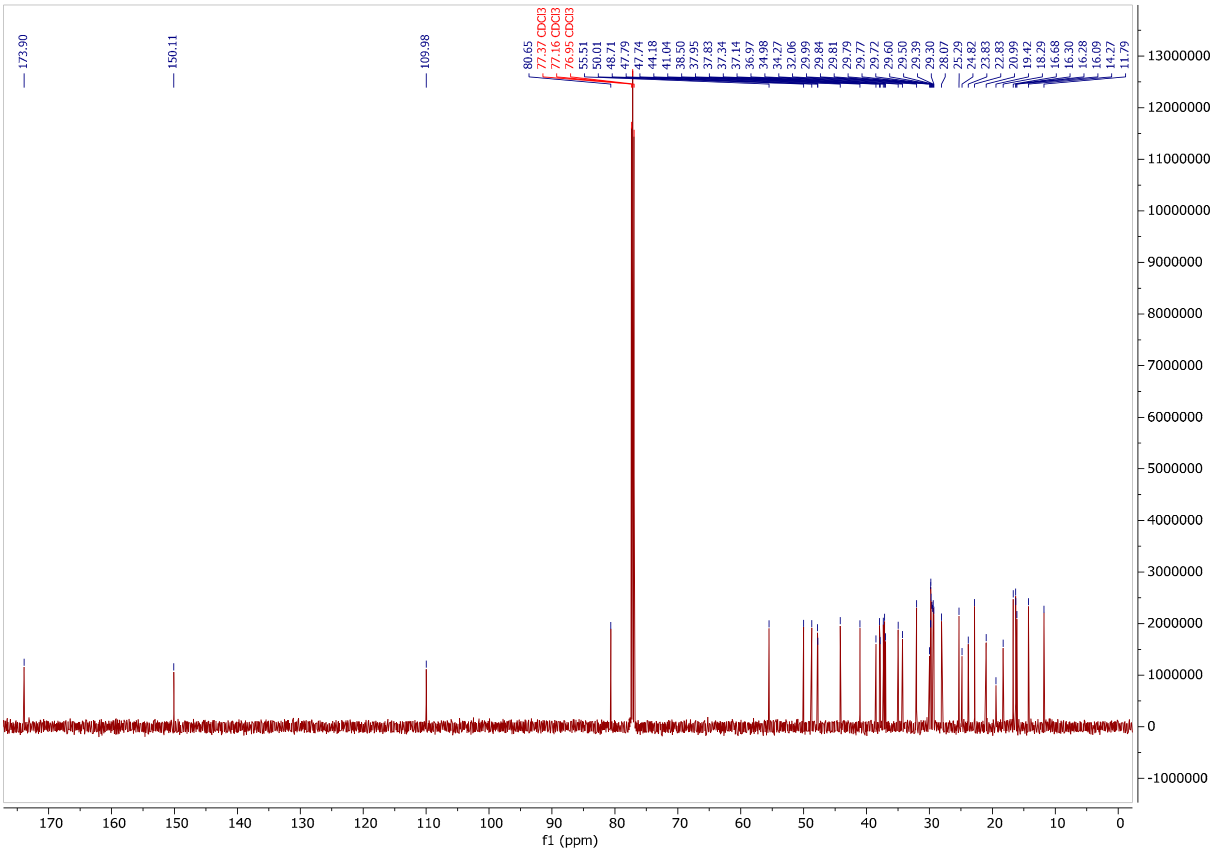

Figure S13. ^{13}C NMR ($CDCl_3$, 150 MHz) of 16-hydroxylupeol-*O*-palmitate

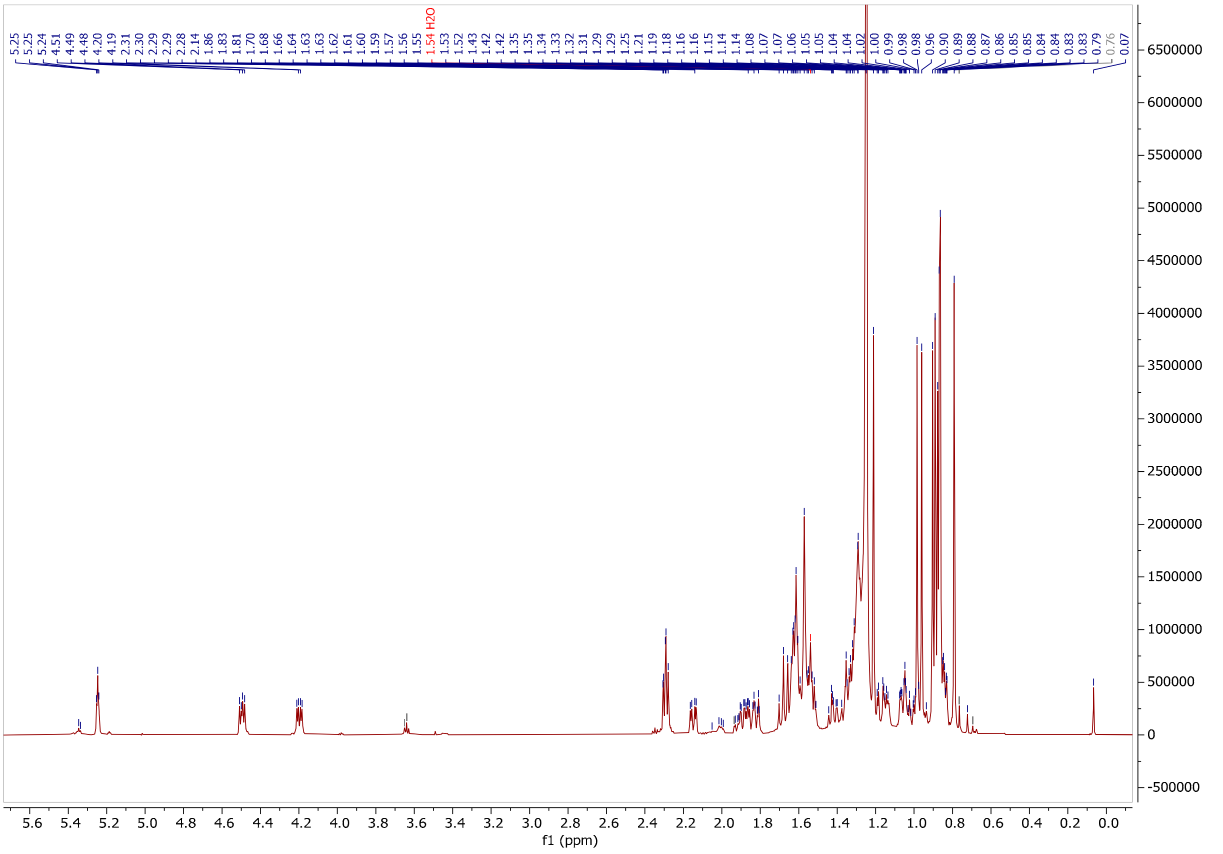

Figure S14. ^{1}H NMR ($CDCl_3$, 600 MHz) of maniladiol palmitate

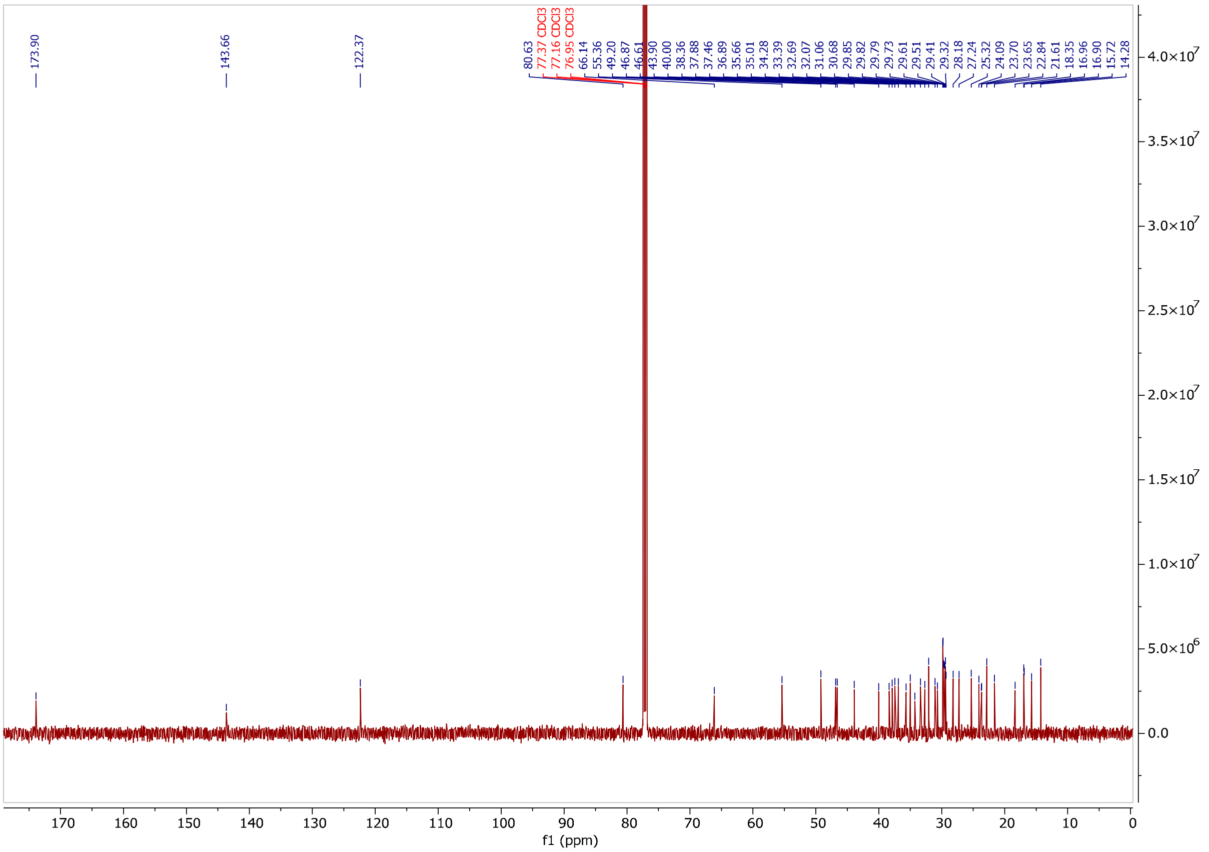

Figure S15. ^{13}C NMR ($CDCl_3$, 150 MHz) of maniladiol palmitate

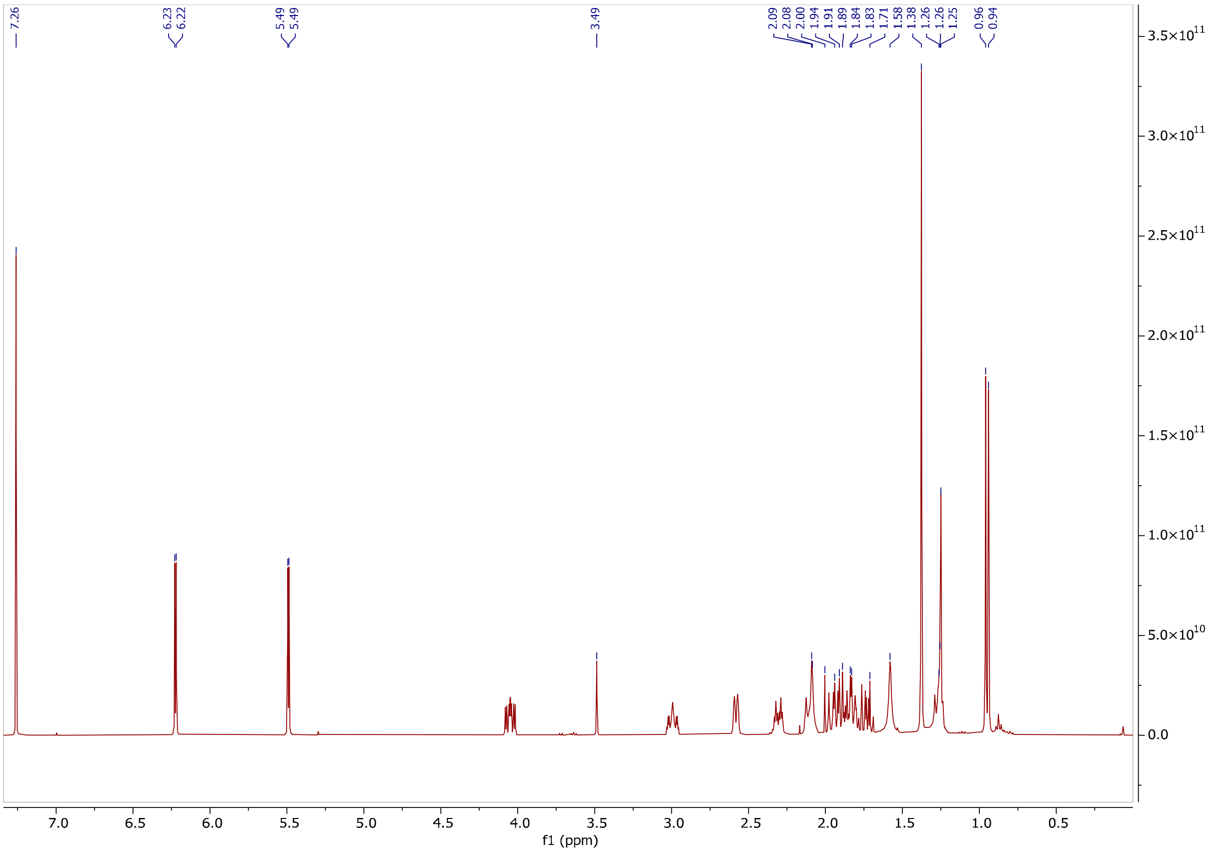

Figure S16. ^{1}H NMR ($CDCl_3$, 600 MHz) of 4α,5α-epoxy-10α,14-dihdyro-1-epi-inuviscolide

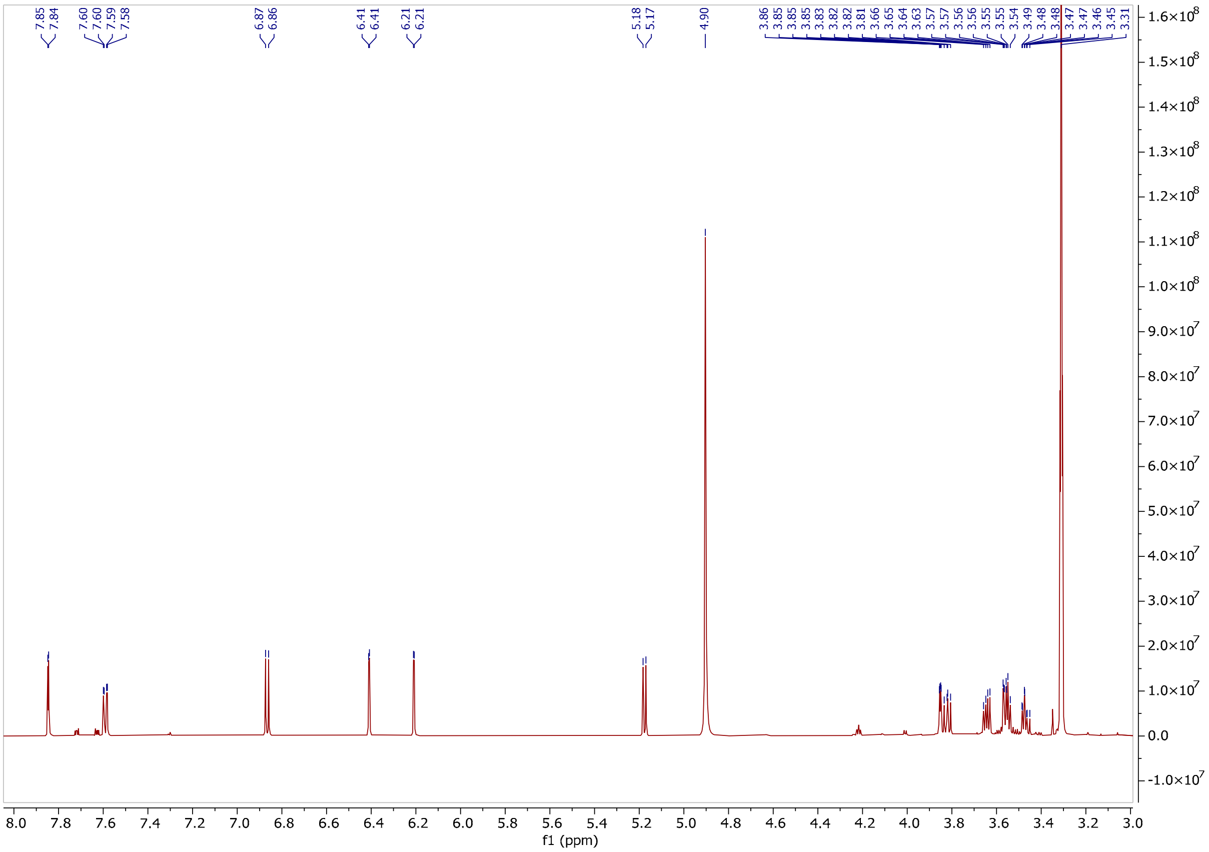

Figure S17. ^{1}H NMR (CD_3OD, 600 MHz) of hyperoside

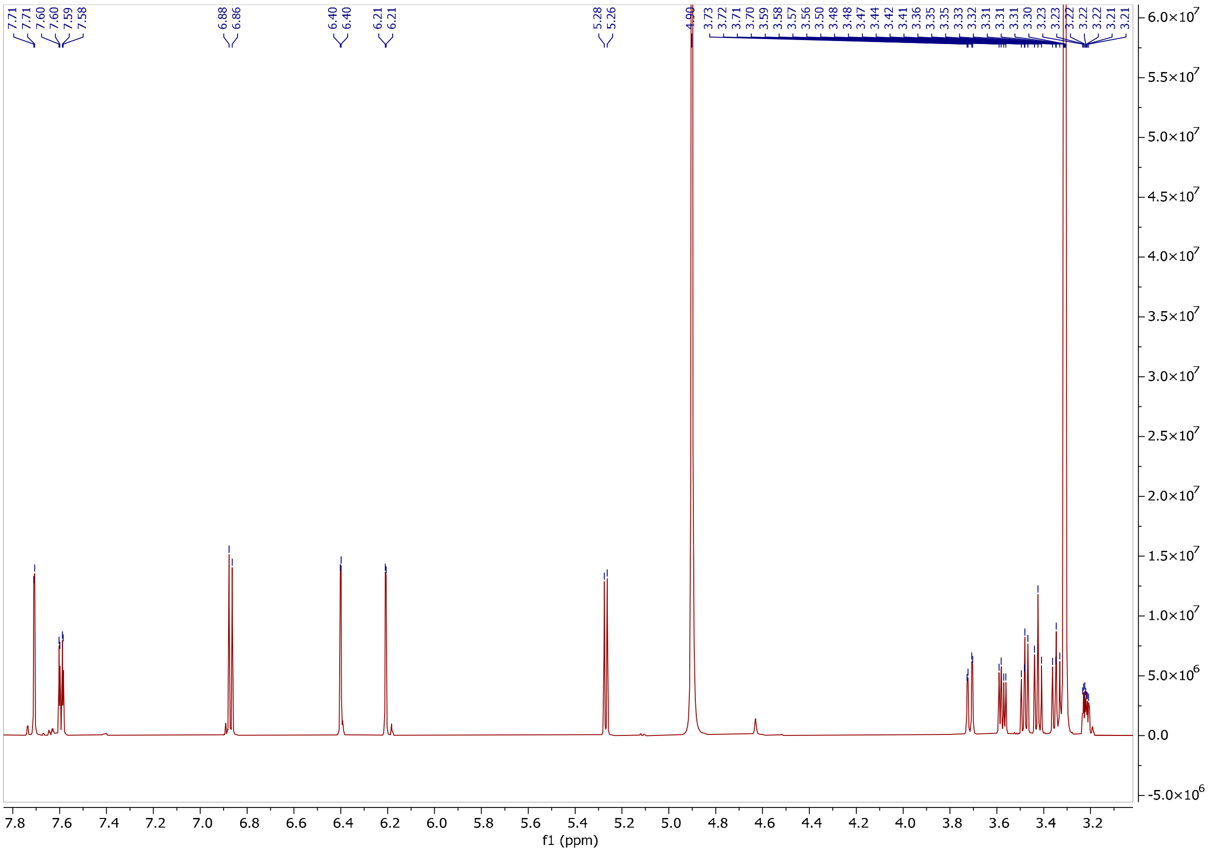

Figure S18. ^{1}H NMR (CD_3OD, 600 MHz) of isoquercetin

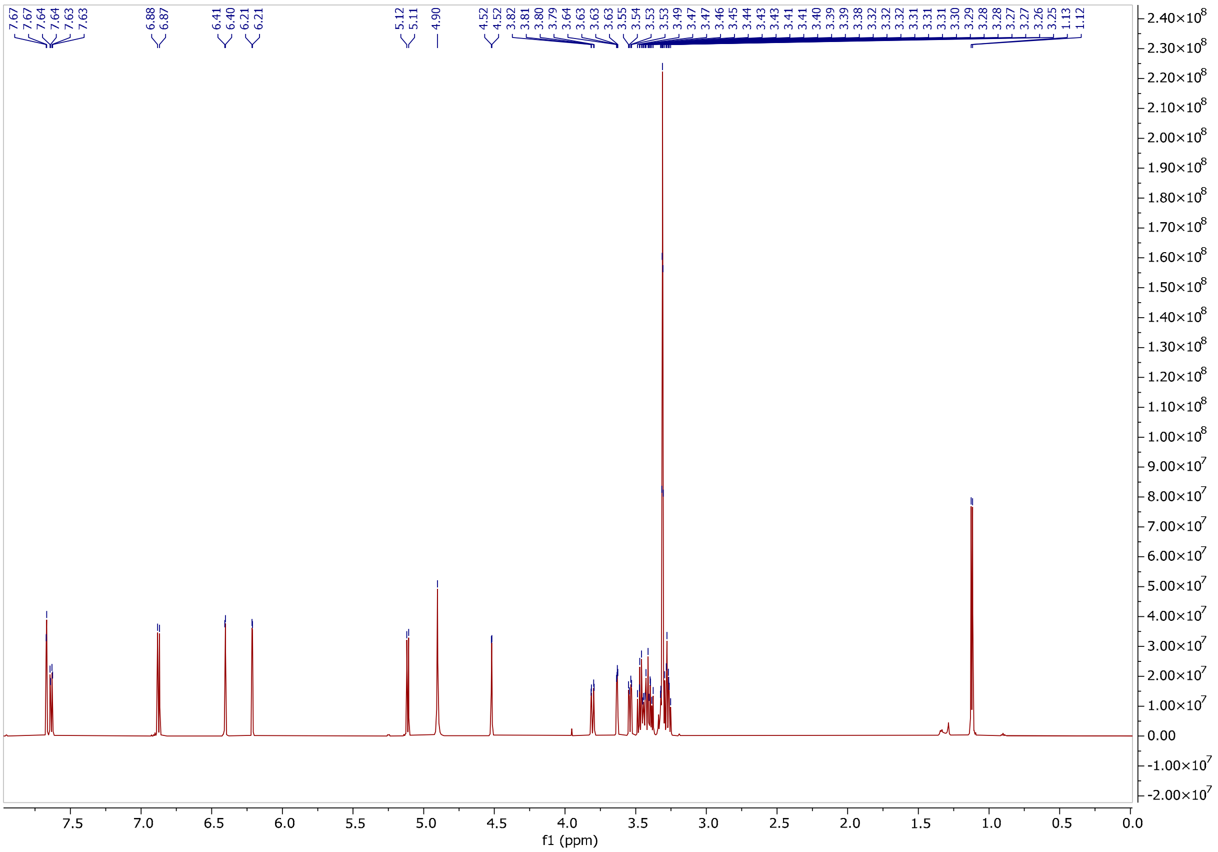

Figure S19. ^{1}H NMR (CD_3OD, 600 MHz) of rutin

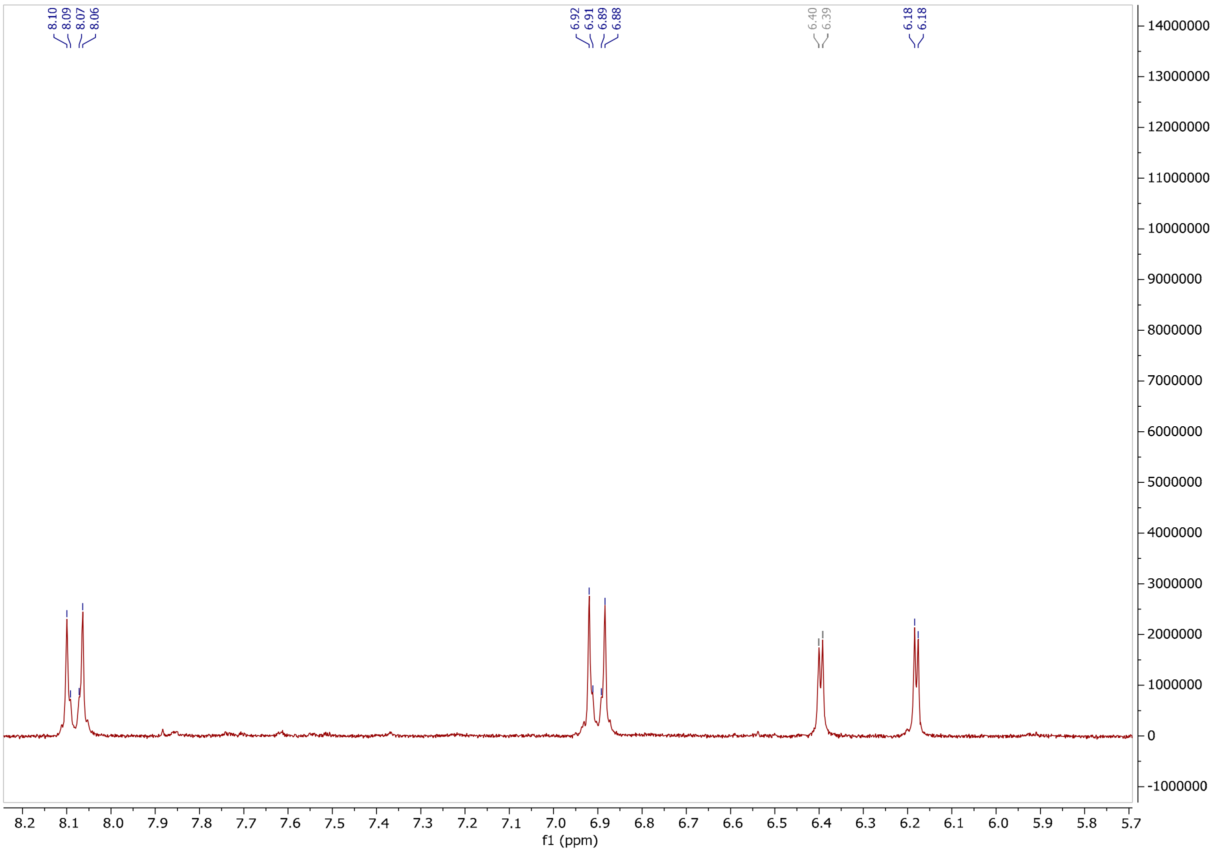

Figure S20. ^{1}H NMR (CD_3OD, 600 MHz) of kaempferol

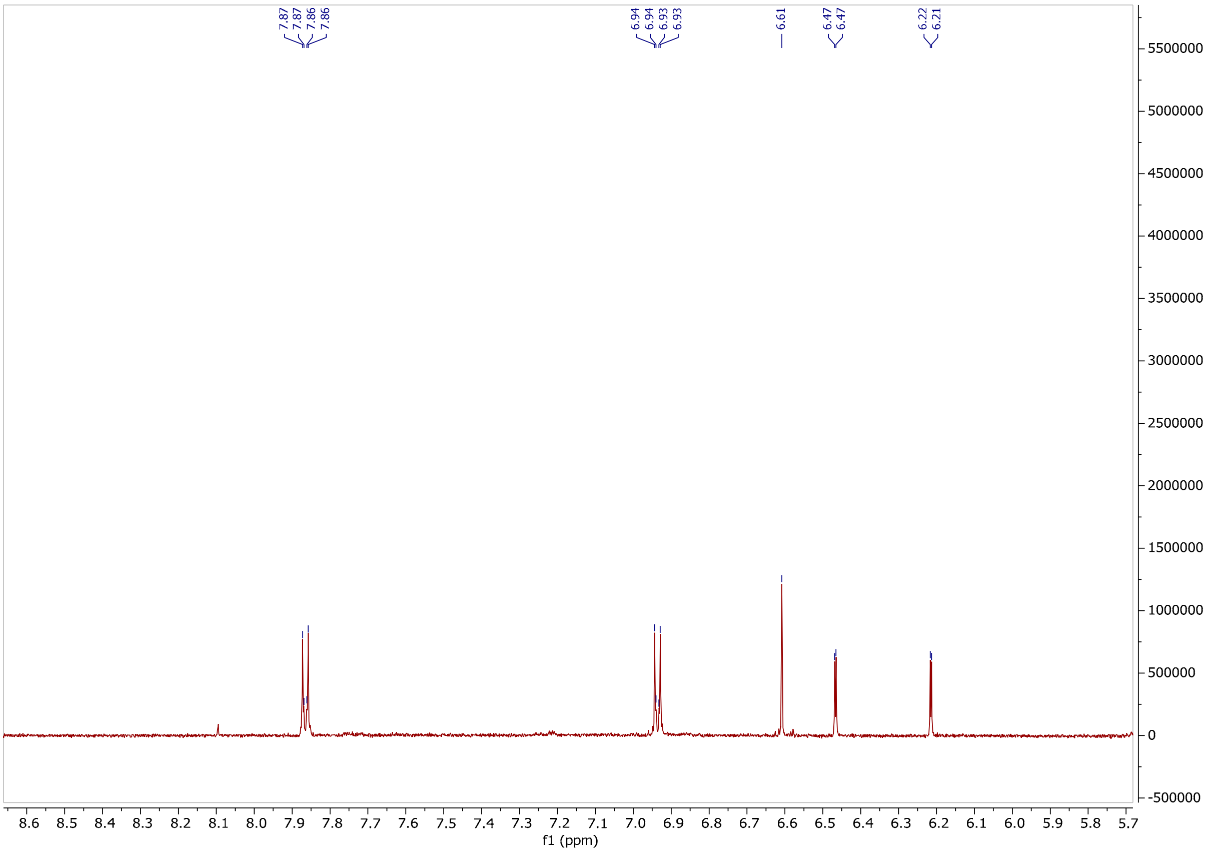

Figure S21. ^{1}H NMR (CD_3OD, 600 MHz) of apigenin

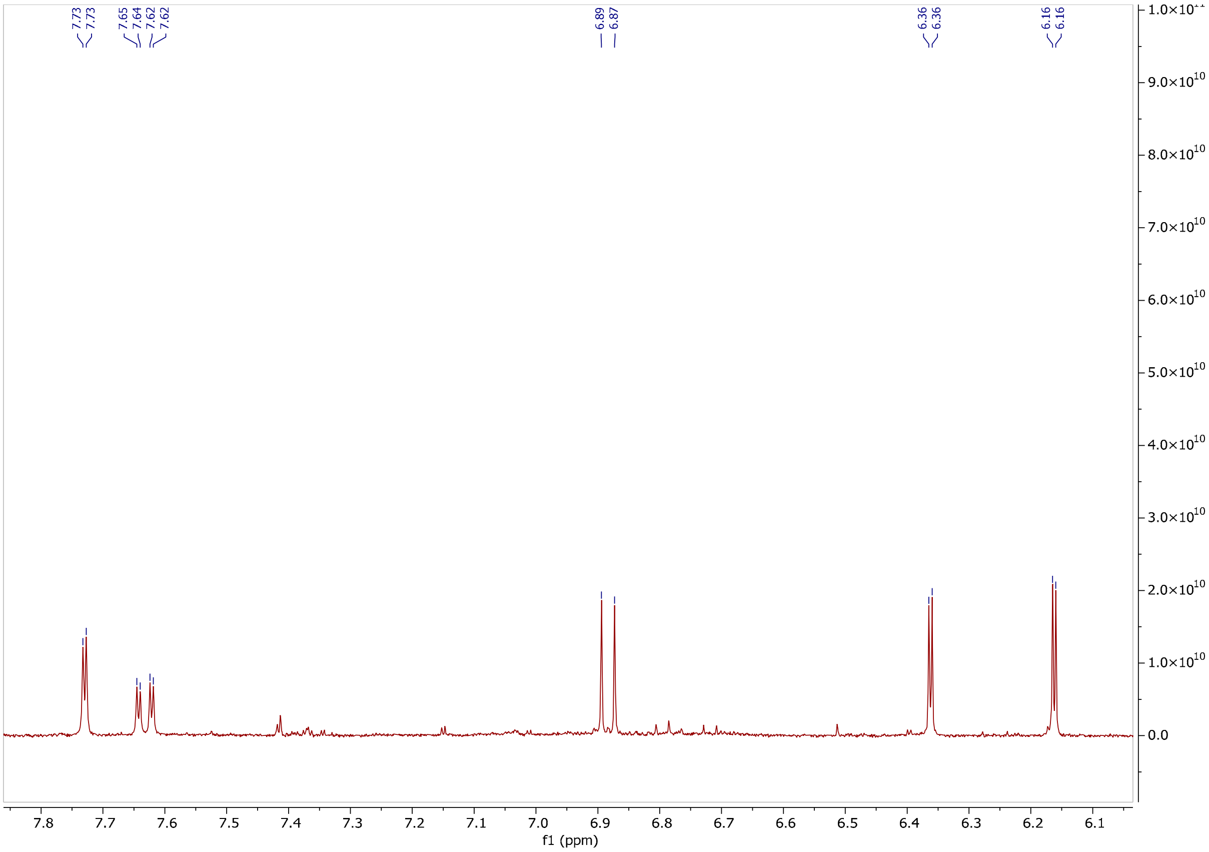

Figure S22. ^{1}H NMR (CD_3OD, 600 MHz) of quercetin

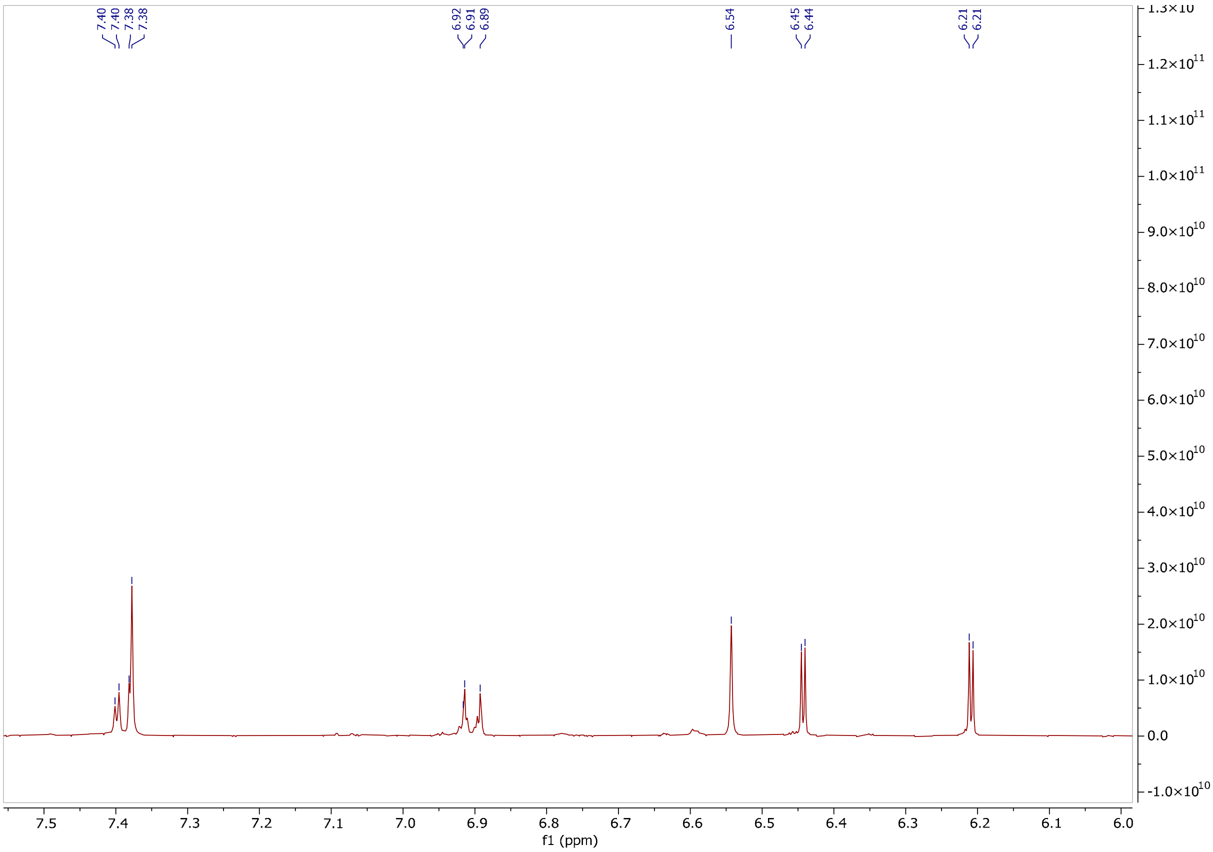

Figure S23. ^{1}H NMR (CD_3OD, 600 MHz) of luteolin

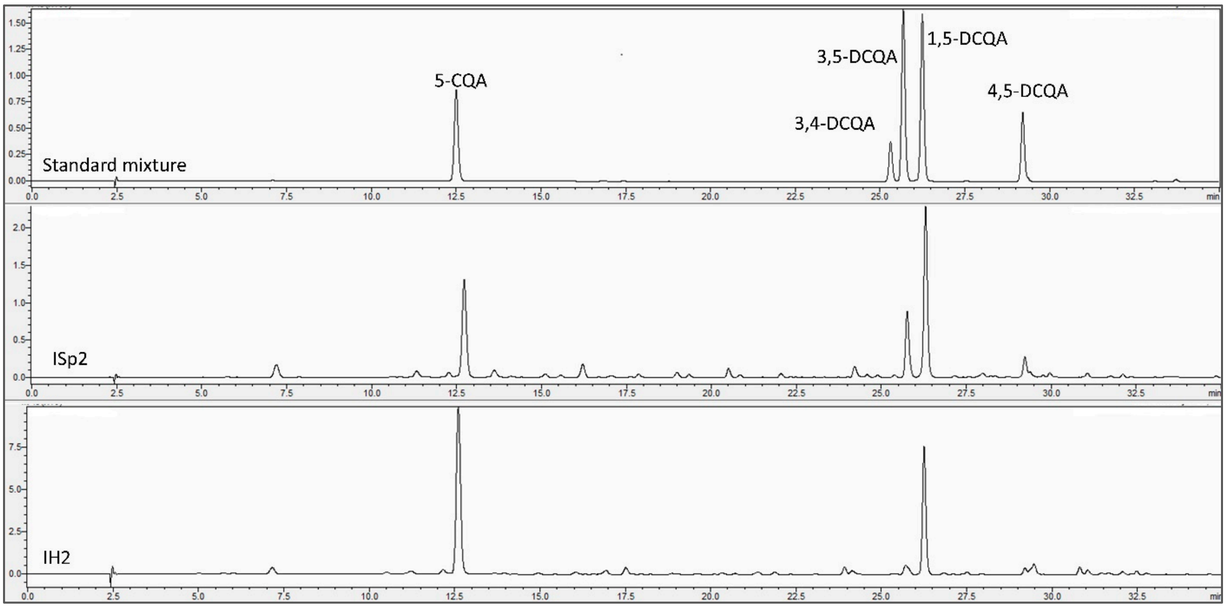

Figure S24. HPLC chromatogram of *I. helenium* and *I. spiraeifolia* methanol extracts (**IH2** and **ISp2**)) and a standard mixture at 320 nm, 5-CQA–chlorogenic acid, DCQA–dicaffeoylquinic acid
